# Decoding the Role of Familial Parkinson’s Disease-Related Genes in DNA Damage and Repair

**DOI:** 10.14336/AD.2022.0216

**Published:** 2022-10-01

**Authors:** Yao-Lin Li, Zhong-Xuan Wang, Chang-Zhou Ying, Bao-Rong Zhang, Jia-Li Pu

**Affiliations:** Department of Neurology, Second Affiliated Hospital, School of Medicine, Zhejiang University, Hangzhou, Zhejiang, 310009, China

**Keywords:** Parkinson's disease, pathogenesis, Nuclear, DNA damage, DNA repair

## Abstract

Parkinson's disease (PD) is a neurodegenerative disease characterized by the degeneration of midbrain substantia nigra pars compacta dopaminergic neurons and the formation of Lewy bodies. Over the years, researchers have gained extensive knowledge about dopaminergic neuron degeneration from the perspective of the environmental and disease-causing genetic factors; however, there is still no disease-modifying therapy. Aging has long been recognized as a major risk factor for PD; however, little is known about how aging contributes to the disease development. Genome instability is the main driving force behind aging, and has been poorly studied in patients with PD. Here, we summarize the evidence for nuclear DNA damage in PD. We also discuss the molecular mechanisms of nuclear DNA damage and repair in PD, especially from the perspective of familial PD-related mutant genes. Understanding the significance of DNA damage and repair may provide new potential intervention targets for treating PD.

Parkinson's disease (PD) is one of the most common neurodegenerative disorders worldwide. Its prevalence increases with age, affecting 4% of the population aged >80 years and far greater than 1% of the population aged >60 years [1, 2]. The key pathogenic hallmarks of PD are the degeneration of dopaminergic neurons in the nigrostriatal pathway and the buildup of Lewy bodies. In addition to the motor symptoms caused by the progressive loss of dopaminergic neurons, patients with PD frequently exhibit various non-motor symptoms, including autonomic dysfunction, insomnia, depression. In most cases, the cause of the disease remains unclear. However, it is currently believed that PD results from the interplay between genetics and the environment [3]. Most cases of PD are sporadic and are related to exposure to environmental chemicals, traumatic brain injury, and lifestyle habits [4, 5]. However, specific gene mutations have been identified in approximately 5-10% of cases, including mutations in *α-synuclein (SNCA)*, *Parkin (PARK2), phosphatase and tensin homolog-induced kinase 1* (*PINK1*), *Parkinsonism associated protein deglycase (DJ-1)* and *leucine-rich repeat kinase 2* (*LRRK2*) [6, 7]. Sporadic and familial PD share common neurodegenerative molecular pathways, including α-syn protein homeostasis, mitochondrial dysfunction, oxidative stress, calcium homeostasis, and neuroinflammation [8-10]. In addition to these mechanisms, as a major driver of aging, DNA damage has drawn increasing attention on how it contributes to the pathogenesis of PD [11-14].

The nuclear genome (nuDNA) is a primary source of genetic information. Although nuDNA is constantly damaged by multiple exogenous and endogenous stimuli, the well-established DNA damage response (DDR) and repair mechanism help maintain genome integrity. The brain consumes the most oxygen in the human body at rest, and because of this high oxygen consumption, neuronal cells create more reactive oxygen species, which can cause oxidative DNA damage. In addition, the physiological activities of neurons, including transcription and replication, can cause DNA double-strand breaks (DSBs) [15]. Many harmful substances in the environment also pose a threat to DNA integrity. Cells developed various DNA repair pathways in response to different forms of DNA damage, thereby preserving genomic integrity [16]. For example, helix twisting damage is mainly repaired via nucleotide excision repair (NER). Base mismatches and short deletions/insertions are repaired via mismatch repair. Oxidative damage and small alkylation products are repaired via base excision repair (BER). Repair of DNA single-strand breaks (SSBs) shares a common pathway with BER. Although infrequent, DSBs, whether unrepaired or incorrectly repaired, have serious adverse effects on cell survival and are therefore considered the most severe type of DNA damage [17]. DSBs in proliferating cells can be repaired via homologous recombination (HR), a precise repair mechanism using sister chromatids as a template, whereas in mitotic cells, non-homologous end joining (NHEJ), which is error-prone, is the main repair pathway [18]. Mature neurons are mainly repaired by NHEJ; thus, DSBs may be particularly harmful to neuronal function and survival [19, 20].

Once nuDNA is damaged, cells can immediately initiate DDR, a process that includes DNA damage recognition, initiating a cell signaling cascade that promotes nuDNA repair [21]. The enzymes that coordinate downstream DDR signals include ataxia telangiectasia mutated (ATM), ataxia telangiectasia and Rad3-related (ATR), DNA-dependent protein kinase (DNA-PK), and the poly (adenine dinucleotide phosphate-ribose) (PAR) polymerase (PARP) family. DNA-PK is recruited to DSBs by Ku and activated, facilitating NHEJ [22] [23]. ATM is recruited to damage foci and is activated by the MRE11-RAD50-NBS1 (MRN) complex [24, 25]. ATR is recruited to SSB foci via an ATR-interacting protein [26]. PARP1 recognizes multiple types of DNA damage and, upon activation recruits a large number of DNA repair proteins by generating PAR chains [27]. In addition to nuDNA damage and repair, these pathways are involved in various biological processes, including cell cycle control, replication, transcription, and cell apoptosis[28].

In the following sections, we discuss how nuDNA damage and repair are related to PD and how familial PD-related mutations are involved in DNA damage and repair.

## 1. Evidence of nuDNA damage in PD

Several studies have confirmed that nuDNA damage is significantly increased in the brains of patients with PD. 8-hydroxyguanine (8-OHG) is a marker of oxidative damage in nuDNA. A study comparing nuDNA modification in different brain regions found that the level of 8-OHG was increased in patients with PD, and the substantia nigra had the most notable increase in 8-OHG levels [29]. Moreover, patients with PD have higher levels of oxidative DNA damage products in the cerebrospinal fluid and peripheral serum [30]. The levels of phosphorylated ATM and its downstream targets, such as phospho-γH2AX and p53, were increased in the brains of patients with PD, suggesting activation of DDR [31]. More direct evidence was provided by a study that compared DNA strand breaks in patients with PD and age-matched controls. Interestingly, the brains of patients with PD accumulated more DSBs and SSBs, and this difference was particularly prominent in the midbrain [32]. One potential reason for the increase in DNA damage in the midbrain is that the metabolism of dopamine in the substantia nigra produces various toxic molecules. Not only does DNA damage accumulate, but the DNA repair capacity is also impaired in patients with PD. Fibroblasts extracted from patients with idiopathic or genetic PD exhibit defects in NER capacity after exposure to ultraviolet C [33]. A study on the correlation between DNA repair gene polymorphisms and PD risk revealed that *apurinic/apyrimidinic endonuclease 1, x-ray repair cross-complementing group 1 (XRCC1), and XRCC3* DNA repair gene variants could increase the risk of PD [34]. A case-control study demonstrated that single nucleotide polymorphisms in the *BER* gene are not associated with the risk of developing PD; however, they can increase the risk of PD in individuals exposed to pesticides [35]. A recent study also suggested that an imbalance in the BER pathway might be an important factor driving neuronal degeneration. With age, incomplete or inefficient BER can lead to the accumulation of toxic BER intermediates, which were found to drive neurodegeneration in a PD nematode model [36].

## 2. Specific defects in nuDNA damage repair pathways cause damage to the dopamine axis

Disruption of the dopaminergic axis in mice can be caused by loss of function of particular DNA repair proteins. ATM is a protein kinase involved in DNA repair that regulates DDR signaling [28]. In one study, ATM-deficient mice were created, and extensive degeneration of dopaminergic neurons in the substantia nigra striatum was observed, accompanied by a reduction in dopamine transporter (DAT) levels in the striatum [37]. The Nijmegen breakage syndrome 1 (NBS1) protein is a component of the MRN complex and is primarily responsible for DSB repair. Mice with NBS1 inactivation in the central nervous system showed a decrease in tyrosine hydroxylase-positive cells in the substantia nigra as well as a decrease in DAT in the striatum [38]. As MRN can activate protein kinase ATM, the neurological deficits in ATM mice are mainly due to DDR failure rather than the cytoplasmic effect of ATM[38]. Excision repair cross-complementation group 1 (ERCC1) is a key DNA repair gene in dopaminergic neurons that can inactivate the NER pathway [39]. ERCC1-deficient mice have pathological manifestations similar to PD, including reduced striatal dopaminergic innervation, higher levels of phosphorylated synuclein, and impaired mitochondrial respiration, and are more susceptible to the PD toxin 1-methyl-4-phenyl-1,2,3,6-tetrahydropyridine (MPTP) [33]. A similar situation was observed in mice with 8-oxoguanine glycosylase (OGG1) deficiency, a DNA oxidative damage repair enzyme. OGG1 knockout mice exhibit pathological manifestations of substantia nigra dopaminergic neuronal loss and symptoms of reduced spontaneous motor movements in old age and are more sensitive to MPTP impairment in young age [40]. Endonuclease 8-like 1 (NEIL1), a DNA glycosylase, has been shown to be involved in contributing to multiple DNA damage repair pathways such as BER and NER [41]. Patients with PD often exhibit non-motor symptoms such as olfactory dysfunction before developing motor impairments [42]. One study found that mice lacking NEIL1 exhibited significant olfactory dysfunction compared with wild-type mice [43]. This suggests that non-motor symptoms of PD are also related to defects in DNA repair. These above mouse models highlight the importance of effective DNA damage repair to reduce the pathological manifestations of PD.

## 3. Familial PD-related mutations and DNA damage and repair

Common familial PD-related mutations include those in *SNCA*, *PARK2*, *DJ-1*, *PINK1*, and *LRRK2*. Exploring whether these genes are involved in DDR and whether their mutations disrupt DDR will help understand the role of DNA damage in PD [14]. In the subsequent sections, we discuss the connection between these genes and DNA damage and their possible roles in the process of DNA repair.

### SNCA

Missense or multiplication mutations in the *SNCA* gene, which encodes α-syn, can result in autosomal dominant familial PD [44]. Studies have also identified a correlation between single-nucleotide polymorphisms in *SNCA* and the risk of sporadic PD [45, 46]. Pathologic α-syn can also activate DDR; overexpression or intracerebral seeding of α-syn caused DDR activation in mice, and this response was weakened by supplementing exogenous antioxidant substances or activating the endogenous antioxidant system [47]. The treatment of primary mouse cortical neurons with α-syn pre-formed fibrils cause DNA damage and PARP1 activation by activating nitric oxide (NO) synthase to produce NO [48]. The activation of PARP1 can enhance the production of pathological α-syn and promote neuronal death via parthanatos [48]. Application of DNA-damaging factors aggravates neuronal degeneration in A53T-α-syn transgenic mice. Following radiography-induced DNA damage, the mice exhibited early dyskinesias, and the number of dopaminergic neurons in the substantia nigra striatum decreased [49]. In addition, the DNA damage repair process in A53T-α-syn mouse embryonic fibroblasts is prolonged [49]. The above studies suggest that the toxic protein α-syn can induce DNA damage by promoting oxidation, ultimately leading to cell death.

Current research is contrasting regarding whether α-syn is important for DNA damage repair. Multiple studies have reported that α-syn is located in the nucleus [50-53]; however, the function of α-syn in the nucleus remains unknown. In paraquat-treated mice, α-syn exhibited increased nuclear localization [50]. By overexpressing α-syn mutations, A30P, and A53T, increased content of α-syn in the nucleus was found [51]. These results indicate that α-syn accumulation in the nucleus may be involved in the pathogenic effects of environmental toxicants and genetic mutations. Regarding the role of α-syn in the nucleus, one study showed that α-syn might function by binding to histones in the nucleus, reducing histone acetylation [51]. However, α-syn overexpression in the nucleus can bind to DNA and induce DSBs, and oxidized or misfolded α-syn can lead to enhanced DNA damage [54]. Another study also indicated that glycated α-syn increases DNA damage by directly interacting with DNA[55]. These findings suggest that an abnormal form of α-syn can induce neuronal genome damage. Interestingly, a previous study demonstrated that α-syn could bind to DNA and promote NHEJ in vitro [56]. The study also found that α-syn colocalized with known DDR components and α-syn knockout mice showed increased DSBs [56]. Recently, an in-depth study was conducted on the interaction α-syn with histones and DNA [57]. The authors found that α-syn specifically interacted with histones, whereas DNA binding was weak and non-specific [57]. Furthermore, phosphorylation mimicking α-syn (S129E) mutant has increased binding affinity for histones than α-syn (wild type) [57]. Based on the above results, it is proposed that histones, rather than DNA are the primary substrates for the regulatory function of α-syn regulatory. In conclusion, the role of α-syn under normal physiological conditions and the role of different α-syn aggregate forms and modifications in the nucleus under pathological conditions warrants further study.

### PINK1/PARK2

PARK2 is an E3 ubiquitin ligase, and PINK1 is a ubiquitin kinase; these enzymes function in tandem to mediate mitophagy to maintain mitochondrial homeostasis [58]. Mutations in *PARK2* and *PINK1* are linked to both familial and sporadic PD [59, 60]. PARK2 also has multiple functions in the nucleus in addition to its involvement in mitochondrial quality control. Interestingly, PARK2 can translocate into the nucleus through an unknown route when the DNA is damaged [61]. Moreover, research has shown that PARK2 can promote DNA damage repair due to ultraviolet rays or oxidative stress [62]. Immunoprecipitation experiments have also demonstrated that endogenous PARK2 in cells can interact with proliferating cell nuclear antigen, a DNA repair protein [62]. Another study found that PARK2 may participate in translesion DNA synthesis by promoting the formation of replication protein A-coated single-stranded DNA and interacting with NBS1 [63]. PARK2 also acts as a transcriptional regulator of genes. PARK2 binds to the promoter and reduces the expression of p53, a versatile protein that participates in DNA repair, cell cycle control, programmed cell death, and other processes [64]. This effect was eliminated by familial *PARK2* mutations [65]. Furthermore, PARK2 which has undergone S-nitrosylation, exhibits reduced binding to p53 [66]. In the brains of patients with PD and pesticide-induced PD mouse models, elevated S-nitrosylated PARK2 is accompanied by an increase in p53 expression [66]. Recently, a study revealed that mitochondrial damage mediates the degradation of breast cancer susceptibility gene 1 (BRCA1), a key protein engaged in HR, via the PINK/PARK2 axis in the nucleus and then induces DSBs [67]. This research revealed the potential association of PARK1/PARK2 with DNA repair from a new perspective. These above results suggest that *Parkin/PINK* may have a neuroprotective effect through involvement in DNA repair.

### DJ-1

DJ-1 is found in the cytoplasm, mitochondria, and the nucleus. It is involved in multiple cellular processes, including oxidative stress and mitochondrial homeostasis [68]. Mutations in *DJ-1* can lead to autosomal recessive early-onset PD [69]. DJ-1 is transferred to the nucleus after 6-hydroxydopamine (6-OHDA) treatment [70]. Increased expression of DJ-1 in the nucleus by tagging the nuclear localization signal in DJ-1 inhibits cell death in response to 6-OHDA, suggesting that DJ-1 in the nucleus plays a protective role [70]. Nuclear translocation of DJ-1 also occurs in neural stem cells in an MPTP-induced mouse PD model [71]. Evidence indicates that DJ-1 could regulate the transcription of multiple genes in the nucleus, including *p53* [72, 73]. A study found that DJ-1 can bind and activate sirtuin 1 (SIRT1), a multifunctional protein involved in multiple physiological processes in cells, such as DNA repair [73]. The decreased SIRT1 activity in DJ-1 knockout cells can be reversed by DJ-1 supplementation [73]. This indicates that DJ-1 regulates DNA repair via SIRT1. In a study of primary alveolar type II cells in emphysema, it was found that DJ-1 interacted with XRCC4-like factor, which is one of the components of the NHEJ connection complex [74]. In addition, the same authors found that *DJ-1* knockout mice showed greater DNA damage when exposed to cigarette smoke than wild-type mice [74]. Another study proposed that DJ-1 can repair DSB-induced DNA glycation damage [75]. Therefore, DJ-1 may be involved in DNA damage repair.

### LRRK2

**Figure 1.**
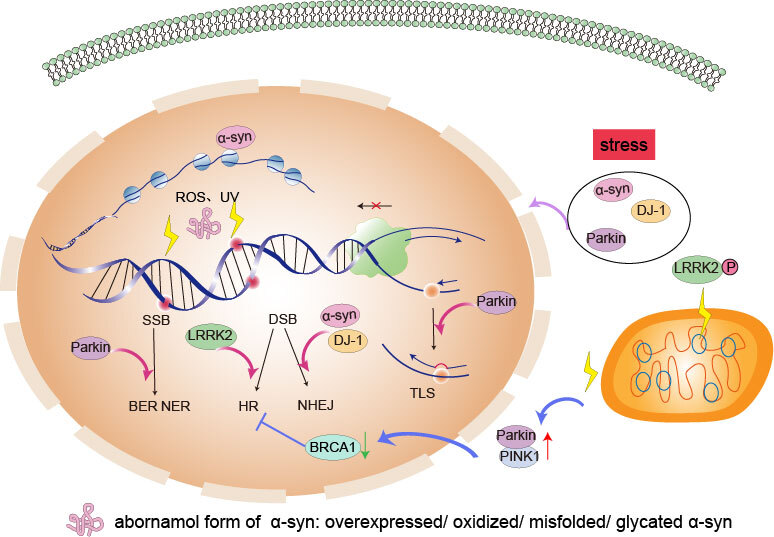
**The possible role of common familial Parkinson’s Disease-related mutations in DNA damage repair**. α-synuclein (α-syn), Parkinsonism associated protein deglycase (DJ-1), and Parkin can translocate into the nucleus under stress conditions. α-syn can interact directly with histones and DNA. Abnormal forms of α-syn in the nucleus, such as overexpressed, oxidized, and misfolded α-syn can cause DNA damage. a-syn has also been reported to be involved in the repair of DNA double-strand breaks (DSBs) via non-homologous end joining (NHEJ) pathway. Parkin is reported to be involved in DNA excision repair caused by ultraviolet rays (UV) and oxidative stress. In addition, Parkin is found to be involved in translesion DNA synthesis (TLS) that bypasses lesion damage when the progression of the replication fork is blocked. DJ-1 is also assumed to be involved in the NHEJ pathway to repair DNA DSBs by a study. Mutations that cause abnormally increased activity of leucine-rich repeat kinase 2 (LRRK2) kinase can cause damage to mitochondrial DNA. LRRK2 inhibitors hinder the homologous recombination (HR) pathway indicating its potential role in DNA repair. Mitochondrial damage can cause DNA DSBs in the nucleus via the phosphatase and tensin homolog-induced kinase (PINK1)/Parkin/breast cancer susceptibility gene 1 (BRCA1) axis.

Mutations in the *LRRK2* gene lead to delayed autosomal dominant PD [76]. Induced pluripotent stem cell-derived neural cells from patients with PD with *LRRK2* mutations exhibited higher levels of mitochondrial (mtDNA) damage than cells from healthy patients without mutation; the increased mtDNA damage caused by *LRRK2* mutations was reversed by genome editing [77]. Another study found that mtDNA damage caused by *LRRK2* mutations is dependent on LRRK2 kinase activity [78]. In addition to affecting mtDNA stability, LRRK2 has been found to promote genome stability in the nucleus. In response to DNA damage, LRRK2 is phosphorylated, interacts with ATM, and mediates cell progression via the murine double minute 2-p53 pathway [79]. By exploring the function of LRRK2 in striatal projection neurons, Chen et al. found that LRRK2 was involved in regulating nuclear morphology and stabilizing the genome. As the mice aged, the γH2AX level, which indicates that DNA damage gradually increased in the striatum of mice, was more pronounced in the striatum of LRRK2-deficient mice [80]. Recently, another study found that LRRK2 inhibition can disrupt the interaction between RAD51 and BRCA2, suppressing HR [81]. The role of LRRK2 in DNA repair mechanism requires further exploration (Fig. 1).

## Conclusion

Previous studies have linked DNA damage with the pathogenesis of various neurodegenerative diseases. Increasing evidence indicates that DNA damage may be a critical step in the pathogenesis of PD; however, whether DNA damage is the driving force behind PD or whether this is induced after the onset of PD needs to be explored. Although various neurodegenerative diseases share defects in DNA damage repair, their pathological manifestations differ. Studying the role of mutations related to the familial forms of these diseases will further our understanding of how defects in DNA damage repair led to these diseases. Some mouse models with specific defects in DNA damage repair show defects in the substantia nigra striatum. The mechanism by which DNA repair defects lead to the degeneration of dopaminergic neurons remains to be elucidated. Furthermore, in mice, DDR activation is one of the many mechanisms of neuronal cell death induced by certain experimental PD neurotoxins, suggesting that defects in DNA damage repair may be a common pathway in the pathogenesis of familial and sporadic PD. DNA damage is also associated with other mechanisms in PD pathogenesis such as the loss of protein homeostasis, inflammation, oxidative stress, and mitochondrial dysfunction [12, 82]. Identifying the link between DNA damage and other mechanisms will aid in our understanding of diseases and enable the design of novel therapies.
